# A Metabolomic and Lipidomic Serum Signature from Nonhuman Primates Administered with a Promising Radiation Countermeasure, Gamma-Tocotrienol

**DOI:** 10.3390/ijms19010079

**Published:** 2017-12-28

**Authors:** Amrita K. Cheema, Khyati Y. Mehta, Oluseyi O. Fatanmi, Stephen Y. Wise, Charles P. Hinzman, Josh Wolff, Vijay K. Singh

**Affiliations:** 1 Department of Oncology, Lombardi Comprehensive Cancer Center, Georgetown University Medical Center, Washington, DC 20057, USA; amrita.cheema@georgetown.edu (A.K.C.); kym8@georgetown.edu (K.Y.M.); jw1@stanford.edu (J.W.); 2 Department of Biochemistry, Molecular and Cellular Biology, Georgetown University Medical Center, Washington, DC 20057, USA; cph51@georgetown.edu; 3 Department of Pharmacology and Molecular Therapeutics, F. Edward Hébert School of Medicine, Uniformed Services University of the Health Sciences, Bethesda, MD 20814, USA; oluseyi.fatanmi@usuhs.edu (O.O.F.); stephen.wise.ctr@usuhs.edu (S.Y.W.); 4 Armed Forces Radiobiology Research Institute, Uniformed Services University of the Health Sciences, Bethesda, MD 20814, USA

**Keywords:** Gamma-tocotrienol, lipidomes, metabolites, nonhuman primates, radiation countermeasure, serum

## Abstract

The development of radiation countermeasures for acute radiation syndrome (ARS) has been underway for the past six decades, leading to the identification of multiple classes of radiation countermeasures. However, to date, only two growth factors (Neupogen and Neulasta) have been approved by the United States Food and Drug Administration (US FDA) for the mitigation of hematopoietic acute radiation syndrome (H-ARS). No radioprotector for ARS has been approved by the FDA yet. Gamma-tocotrienol (GT3) has been demonstrated to have radioprotective efficacy in murine as well as nonhuman primate (NHP) models. Currently, GT3 is under advanced development as a radioprotector that can be administered prior to radiation exposure. We are studying this agent for its safety profile and efficacy using the NHP model. In this study, we analyzed global metabolomic and lipidomic changes using ultra-performance liquid chromatography (UPLC) quadrupole time-of-flight mass spectrometry (QTOF-MS) in serum samples of NHPs administered GT3. Our study, using 12 NHPs, demonstrates that alterations in metabolites manifest only 24 h after GT3 administration. Furthermore, metabolic changes are associated with transient increase in the bioavailability of antioxidants, including lactic acid and cholic acid and anti-inflammatory metabolites 3 deoxyvitamin D3, and docosahexaenoic acid. Taken together, our results show that the administration of GT3 to NHPs causes metabolic shifts that would provide an overall advantage to combat radiation injury. This initial assessment also highlights the utility of metabolomics and lipidomics to determine the underlying physiological mechanisms involved in the radioprotective efficacy of GT3.

## 1. Introduction

The threat of a radiological or nuclear event is a critical concern for all government agencies involved in national security and public health preparedness, as well as for the military [1,2]. As made evident by the BioShield legislation signed into law on 21 July 2004, which provides new tools to improve medical countermeasures protecting Americans against a chemical, biological, radiological, or nuclear (CBRN) attack, the need for new countermeasures that are safe, easily administered, and effective at reducing or eliminating the public health impact of acute, high-dose radiation are urgently needed [3,4]. In the event of radiological or nuclear event, medical care would be needed to treat radiation-exposed victims developing acute radiation syndrome (ARS) [5,6,7]. Acute radiation injury occurs at whole-body doses above 2 Gy, with symptoms growing in severity as the level of radiation exposure increases [5]. A dose range of 2–6 Gy is characterized by the loss of hematopoietic cell regenerative ability, resulting in hematopoietic ARS (H-ARS). In the exposure range of 6–10 Gy, hematopoietic symptoms are present in addition to symptoms caused by significant breakdown of the gastrointestinal (GI) system, resulting in GI-ARS. H-ARS and GI-ARS are recognized as the major sub-syndromes of ARS which can be treated with radiation countermeasures, and our efforts aim to have countermeasures approved for such indications.

Despite significant advances over the past six decades to develop safe, non-toxic, and effective radiation countermeasures for ARS, only two agents have been approved by the United States Food and Drug Administration (US FDA) for human use [3,8,9,10,11]. Further, both these approved agents are radiomitigators, and the US FDA has not approved a radioprotector for the treatment of ARS yet. The dearth of FDA-approved countermeasures has prompted an intensified search for a new generation of radiation countermeasures [12,13,14]. The vitamin E family has eight different isoforms that belong to two groups: four saturated analogues (α, β, γ, and δ) known as tocopherols and four unsaturated analogues called tocotrienols. These eight agents are collectively referred to as tocols and are well known for their antioxidant, neuroprotective, and anti-inflammatory properties [15,16]. Vitamin E regulates peroxidation reactions within the body [17]. Gamma-tocotrienol (GT3) is a potent inhibitor of 3-hydroxy-3-methylglutaryl-coenzyme A (HMG-CoA) reductase. It has been found to be effective for various types of malignancies in animal models and it has also been investigated in healthy human volunteers [18]. During the last 10 years, GT3 has received significant attention and appears to be one of the most encouraging radiation countermeasures among tocols tested to date [17]. GT3 has been shown to be a radioprotector in the murine model of ARS [19]. Lately, GT3 has been tested as a radioprotector in a pilot study in the NHP model and demonstrated efficacy for improving ionizing radiation-induced cytopenia, neutropenia, and thrombocytopenia in the absence of any supportive care [20]. Currently, we are studying GT3 in NHPs for its efficacy against ionizing radiation-induced H-ARS and GI-ARS, and its mechanism of action. GT3 is being developed as a radioprotector for ARS, which can be administered prior to radiation exposure, following the US FDA Animal Rule since such agents cannot be studied for efficacy using human volunteers [21].

Here, we have studied GT3 induced changes in metabolomic and lipidomic profiles as part of its safety and toxicity testing in the NHP model. We report GT3-induced longitudinal changes in metabolic and lipidomic serum profiles of NHPs using a global metabolomic and lipidomic approach with an ultra-performance liquid chromatography (UPLC) quadrupole time-of-flight mass spectrometry (QTOF-MS) platform. We found few changes in metabolite profiles during the first 24 h following GT3 administration. There were significant changes in profiles of several metabolites over time (1–3 days) that returned to baseline levels over the next 20-day follow-up period. Furthermore, there was a significant overlap of metabolite profiles for the three doses of GT3 that were tested in this study. The findings from metabolic profiles were consistent with complete blood count (CBC) and blood chemistry analyses of the same set of animals post GT3 administration. Taken together, these results suggest that GT3 administration does not have adverse consequences on overall metabolism and is safe for use as a potential radiation countermeasure.

## 2. Results

### 2.1. Identification of Metabolite Signatures of GT3 Administration in NHPs

**Figure 1:**
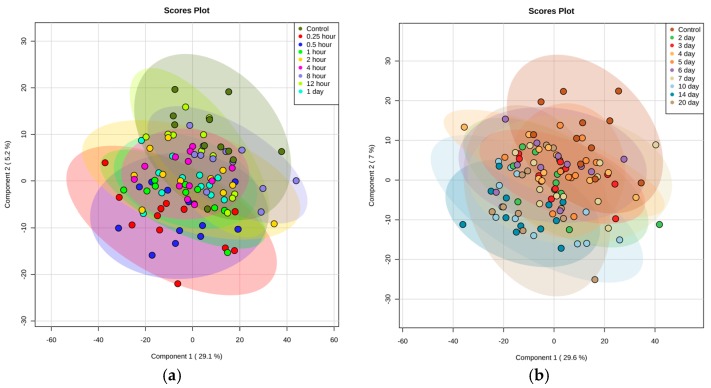
Partial least squares-discriminant analysis (PLS-DA) plots representing biomarkers separation. Multivariate analysis showing metabolic profiles across all Gamma-tocotrienol (GT3)-treated nonhuman primates (NHPs) separated in two time intervals: (**a**) short-term (first 24 h) with R^2^ = 0.52 and Q^2^ = 0.19, and (**b**) long-term (2 days to 20 days) with R^2^ = 0.43 and Q^2^ = 0.0.07. Both groups demonstrate minimal separation, indicating no significant changes amongst groups treated with GT3. Representative data from metabolomics Electrospray Ionization (ESI) positive mode.

Untargeted metabolomic and lipidomic profiling was performed for a total of 208 serum samples obtained from 12 NHPs (four per dosage group) in this pilot study. All NHPs were included in the analysis since there were no outliers. A total of 3667 features in the Electrospray Ionization (ESI) positive mode and 4914 features in the ESI negative mode were detected for metabolomics (Acquity BEH C18 column) while a similar analysis of lipidomics (Acquity CSH C18 column) yielded 1573 features in the ESI positive and 622 in ESI negative mode. Scores plots (Figure 1) were analyzed to evaluate group separation resulting from inherent differences in metabolite profiles of NHPs that received 9.375, 18.75, and 37.5 mg/kg of GT3 as compared to the pre-administration samples. Partial least squares-discriminant analysis (PLS-DA) plots of the negative ionization compounds and lipids are presented in Supplementary Figure S1. The coefficient of variation (CV) values of internal standards for quality control samples (QCS) as well as the total ion chromatogram (TIC) overlays have been presented as Supplementary Figure S2. We asked if GT3 administration induced changes in profiles in the short-term (first 24 h) (Figure 1a) and/or if overall metabolite profiles changed over the long-term (2 days to 20 days) (Figure 1b). Each time point following GT3 administration was defined as an independent class for multivariate analysis. Principal component analysis did not yield separation for any of the models. PLS-DA multi-class models including two components yielded R^2^ = 0.52 and Q^2^ = 0.19 for the short-term group and R^2^ = 0.43 and Q^2^ = 0.07 for the long-term group. There was a significant overlap between the treated groups with the pre-injection samples for time points monitored within the first 24 h, demonstrating that GT3 administration induced minimal changes in overall metabolite profiles. There was a modest separation of treated groups for later time points (1–3 days). Additionally, there was a significant overlap between groups for different doses of GT3. This suggests that a sample size of four may not be large enough to observe dose-dependent metabolic changes when the difference in GT3 concentration is only two-fold. Thus, we determined the mean for results obtained with three different doses of GT3 at each time point to compare with pre-drug injection samples.

**Figure 2:**
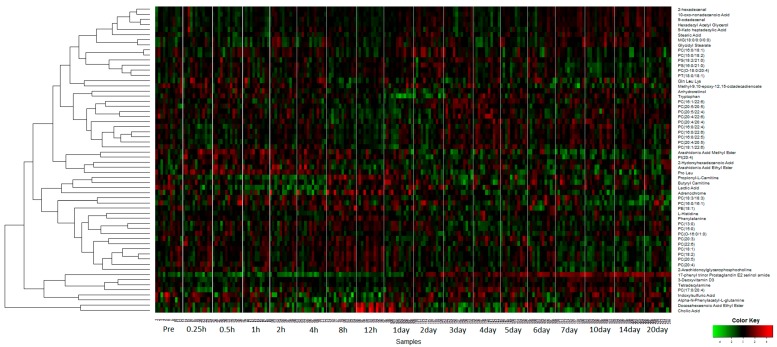
Heatmap illustration of validated metabolites showing transient changes in relative abundance. A total of 61 biomarkers with significant changes (adjusted *p* < 0.05) were validated by tandem mass spectrometry. Hierarchical clustering was used to visualize time and/or dose dependence upon GT3 administration.

Next, chemical formula calculations for 2323 significantly dysregulated metabolites (using ANOVA, adjusted *p* < 0.05) were performed via accurate mass based search and the identity of 61 metabolites was confirmed using tandem mass spectrometry (Supplementary Table S1). We used hierarchical clustering to visualize time and/or dose dependence of these validated metabolites, as a heat map (Figure 2). While there were no appreciable changes in relative abundance within 24 h, we observed transient changes in abundance 1–3 days post-administration of GT3, and the endogenous levels returned to normal over the extended time course.

**Figure 3:**
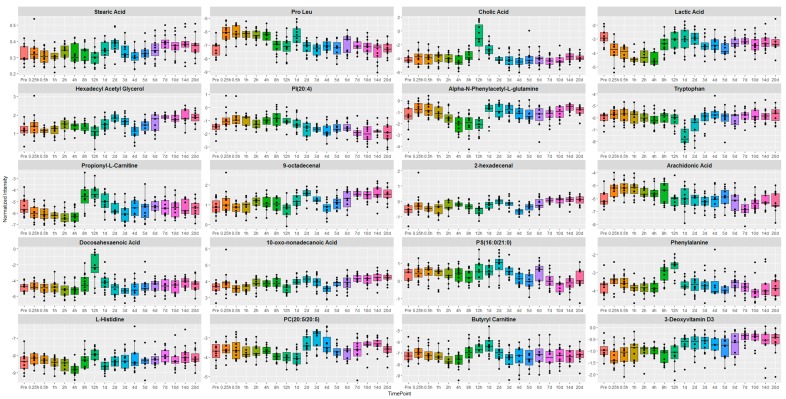
Representative box and whisker plots. Visualization of top 20 biomarkers as box and whisker plots with log transformed normalized intensity along *Y*-axis and time point on *X*-axis. Most biomarkers showed moderate changes in the 12 h to 3 day time points, with stabilization occurring from day 3 onward.

**Figure 4:**
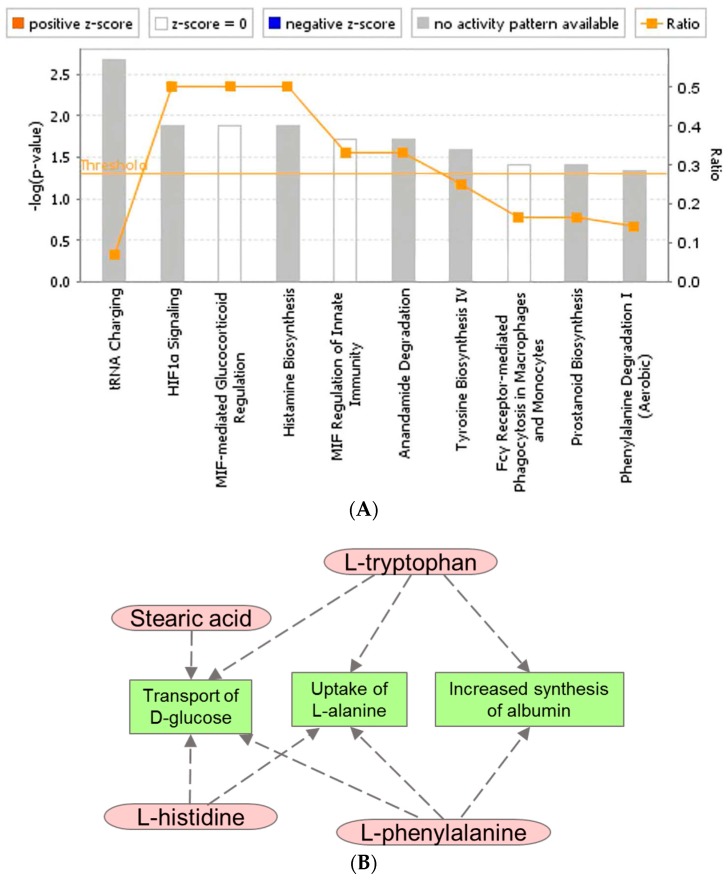
Ingenuity pathway analysis (IPA). Pathway enrichment analysis was performed on validated metabolites. IPA yielded information on 14 of the total validated metabolites. (**A**). Bar graph representing top canonical pathway implicated in these metabolites. (**B**) Network map visualizing representative implicated pathways of key validated biomarkers.

The relative abundance pattern of the top 20 dysregulated metabolites were visualized as box and whisker plots (Figure 3). These metabolites were selected using a hierarchical clustering algorithm, performed using the Random Forests module in Metaboanalyst 3.0 (Xia Lab, McGill University, Montreal, QC, Canada). As stated above, metabolic profiles did not show appreciable change in the first 24 h for all three doses. Changes for endogenous levels were apparent during days 1–3 for metabolites including vitamin D3, phenylalanine, lactic acid, and arachidonic acid and reverted back to near normal (baseline) levels over the 20-day follow-up period. Pathway enrichment analyses of the 61 validated biomarkers, using Ingenuity Pathway Analysis (IPA, Qiagen, Germantown, MD, USA) yielded results for 14 of these biomarkers (Figure 4). The top three canonical pathways implicated in this analysis were tRNA Charging (*p* = 1.44 × 10^−3^), HIF-1α Signaling (*p* = 1.16 × 10^−2^) and Histamine Biosynthesis (*p* = 1.16 ×10^−2^). In particular, Histidine, Phenylalanine, and Tryptophan were linked to amino acid metabolism and transport (decreased uptake of Alanine, *p* = 4.77 × 10^−8^). These biomarkers were also implicated, with Stearic Acid, in carbohydrate metabolism and transport (decreased transport of Glucose, *p* = 9.62 × 10^−7^). Broadly speaking, most of the pathways converged on amino acid metabolism, molecular transport, and cell signaling.

However, these changes did not persist over the entirety of the study. Levels of Histidine, Lactic Acid, Phenylalanine, Stearic Acid, PC(O-18:0/20:4), Propionyl-l-Carnitine, Cholic Acid, α-*N*-Phenylacetyl-l-Glutamine, and PC(16:0/18:1) all marginally increased within 12 h–2 days of GT3 administration, but returned to initial levels from day 3 onward. Other biomarkers in this analysis, like Tryptophan and 10-oxononadecanoic acid, showed modest decreases in the 12 h to 2-days range, but stabilized to normal levels from day 3 onward.

### 2.2. Effects of GT3 on CBC and Blood Biochemistry Parameters

We have investigated CBC and blood biochemistry parameters in samples obtained from GT3-treated animals at various time points prior to or after GT3 administration to NHPs. Three different doses of GT3 were injected to four animals each.

**Figure 5:**
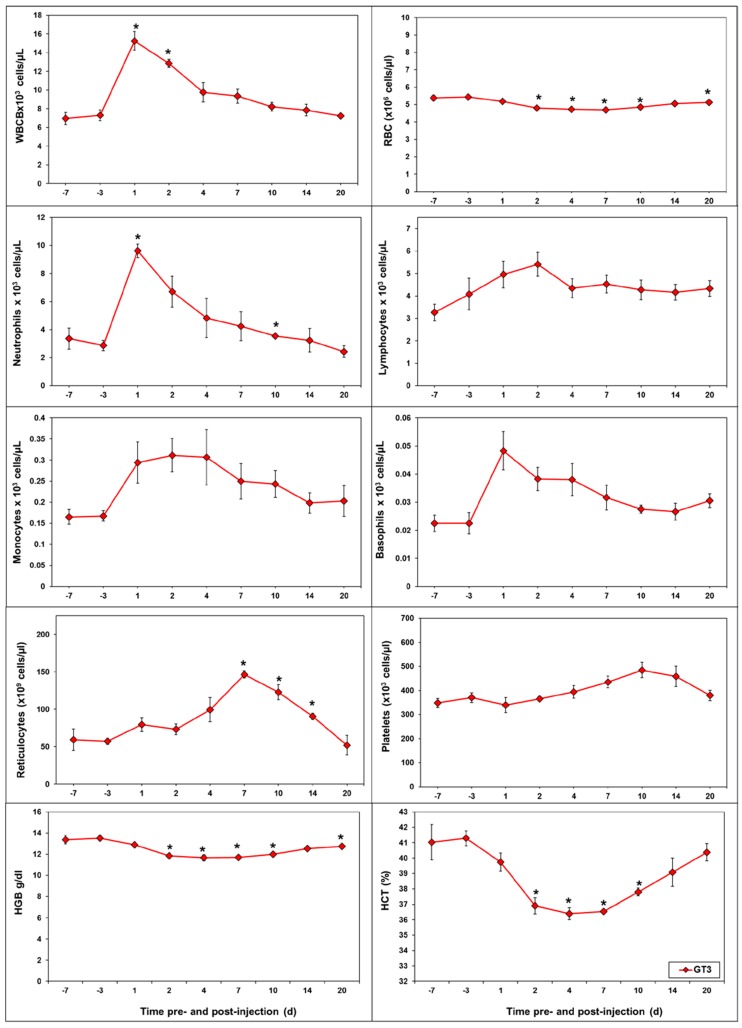
GT3-induced changes in complete blood counts as well as differential counts. GT3 was injected (subcutaneous, sc) to NHPs. Blood was collected at various time points. Cells were counted using a Bayer Advia-120 cell counter. The data for each time point is presented as the mean ± standard error. The difference between GT3-treated doses (average of all doses) and their respective baseline measurements (average of pre-irradiation values on SD-7 and -3), when significant (*p* ≤ 0.05), is indicated with “*”.

**Figure 6:**
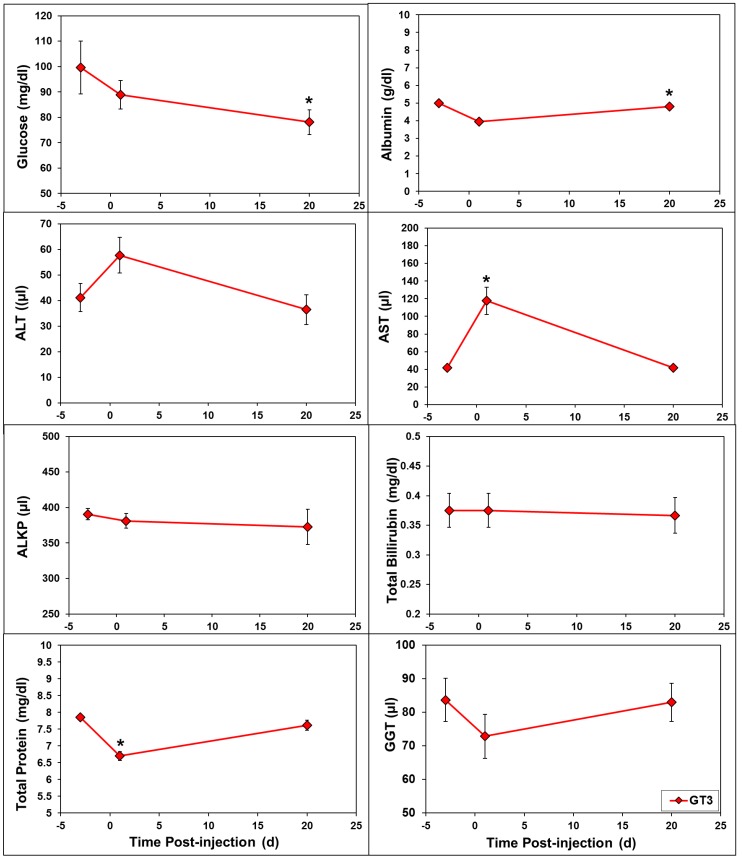
GT3-induced changes in serum biochemistry. GT3 was injected (sc) to NHPs. Blood was collected at various time points for serum chemistry analysis. The difference between GT3-treated doses (average of all doses) and their respective baseline measurements taken on SD-3, when significant (*p* ≤ 0.05), is indicated with “*”.

The different doses of GT3 largely followed the same pattern throughout the study. This suggests that the sample size (*n* = 4) may not be large enough to observe dose-dependent changes in the hematological profile when the difference in GT3 concentration is only two-fold. As a result, we have presented the mean of all three doses of GT3 and comparing pre- and post-irradiation values. Among CBC parameters, white blood cells (WBC) and neutrophils were affected during the 1–2 day window after GT3 injection (Figure 5). Platelets and red cell associated parameters (RBC, reticulocytes, HGB, and HCT) were affected beyond day 2 post-GT3 injection. While there was an increase in the levels of platelets and reticulocytes, we noticed a decrease in the levels of HGB and HCT. It is important to note that both neutrophils and WBC which are important CBC components for radioprotection, were increased after GT3 injection. Out of eight blood chemistry parameters presented in Figure 6, albumin, total protein, and AST were found to be altered at 24 h following GT3 administration.

### 2.3. Preliminary Effects of Varying GT3 Doses or Gender on Metabolomic and Lipidomic Profiles

Next, we conducted a preliminary investigation into potential GT3 dose and gender effects on the resultant metabolite profiles. As discussed above, since we did not see significant variation across samples obtained from NHPs that received different doses of GT3, we hypothesized that dose was not a significant variable. First, in order to examine dose effects, we segregated NHP’s into the three dose groups for the significantly dysregulated metabolites and plotted the trend lines for each dose (Supplementary Figure S3). In addition, we performed an ANOVA comparison for the three doses at each time point (Supplementary Table S2). We found that of the 20 biomarkers in Figure 3, only two showed a significant adjusted *p*-value for one specific time point each (Pro-Leu at day 6 and Alpha-*N*-Phenylacetyl-l-glutamine at day 14). These results lend credence to our assumption that GT3 doses used in this study did not induce dose specific metabolic alterations.

Next, we aimed to determine if there were differences in the metabolomic and lipidomic profiles of male and female NHPs. Principal component analysis did not yield any meaningful separation between males and females. However, a two-component PLS-DA model did show partial separation between males and females, with some overlap (Supplementary Figure S4A) with modest quality of the model. The samples were further annotated with time-point specific identification to ensure that the modest group separation was not a time effect. Given the minimal separation of time within genders, we identified three time points (post-GT3 administration) that separated out against the control NHPs for performing a sub-set analysis to examine gender effects on metabolic alterations. We analyzed samples from the control group and 8-h, 1 day and 3 day treatment groups using a two-component PLS-DA model with modest separation (24.3% on PC1 and 17.6% on PC2) suggesting marginal effects caused by gender (Supplementary Figure S4B). Next we interrogated the difference in trend lines for the top 20 validated metabolites to see gender-specific changes. The results were visualized as trend plots for the time course of GT3 administration (Supplementary Figure S5). In addition, we performed binary comparisons between male and female at each time point for these metabolites that showed non-significant *p*-value when comparing gender based differences in metabolite abundance for all time points except for 3-Deoxyvitamin D3 at day.

## 3. Discussion

GT3 is a promising radioprotector under advanced development that can be administered prior to radiation exposure for the benefit of military personnel, first responders, and the civilian population [17]. It has shown efficacy in the murine as well as in the NHP model against ^60^Co γ-irradiation. Its radioprotective efficacy in mice has been demonstrated to be mediated through G-CSF and administration of G-CSF, neutralizing antibody to GT3-treated and irradiated mice completely abrogated its radioprotective efficacy [19]. A single administration of GT3, without any supportive care, was equivalent, in terms of improving hematopoietic recovery, to multiple doses of Neupogen and two doses of Neulasta with full supportive care (including blood products) in the NHP model [17,20].

Currently, GT3 is under advanced development as a radiation countermeasure for ARS, and it is imperative to characterize metabolic changes that are induced by GT3 administration that could potentially interfere with the overall physiology of an individual. Pharmacokinetic studies in NHPs have provided crucial insights into the turnover and efficacy of this drug as a radioprotectant [20,22,23,24]. Hence, metabolomic and lipidomic profiling is likely to provide an orthogonal assessment of the safety and efficacy of GT3 administration.

Recently, GT3 has also been studied for modulating microRNA (miRNA) and metabolomes in irradiated NHPs with an objective to identify its efficacy biomarker [25,26]. We have reported the correlation of evolutionarily-conserved miRNAs with the impact of GT3 on the radiation response of NHPs. Serum miRNA levels of miR-30a, miR-126, and miR-375 correlated with the radioprotective efficacy of GT3 [25]. These miRNA in the GT3-treated irradiated NHPs resembled the unirradiated animals. These three miRNAs could be used as biomarker of GT3 efficiency in protecting animals from the impact of irradiation. In a preliminary report from a study conducted in irradiated animals, we have demonstrated that GT3 administration reduced high fluctuations in serum metabolite levels in NHPs exposed to 6.5 Gy ^60^Co total-body radiation, which may suggest beneficial physiological effects on fatty acid synthesis, DNA damage, increased muscle function, amino acid metabolism, and renal function. Though serum sample analysis was only conducted at two time points post-irradiation (12 and 24 h) in addition to the pre-irradiation sample, this preliminary result indicates an overall positive effect of GT3 on animals exposed to ionizing radiation [26].

Exposure to ionizing radiation stimulates a set of complex biological responses including gene expression and protein synthesis that ultimately leads to dysregulation of metabolic processes [27,28]. However, longitudinal metabolic changes caused by GT3 administration have not been delineated. Hence, in the present study, we used a global lipidomic and metabolomic approach to determine changes in serum metabolite profiles in NHPs that were administered GT3 in comparison with the samples collected prior to GT3 injection. We observed no significant changes during the first 24 h after GT3 administration. However, metabolic changes were apparent in GT3-treated animals between 1–3 days, and these changes overlapped between the three doses tested in this study. Four NHPs were in each group with a two-fold increase in doses of GT3; this may not be enough to observe dose-dependent changes. Due to the ethical considerations and other issues associated with NHP studies, it is not easy to have a large sample size for such studies. Strikingly, we observed a transient elevation of metabolites known to have a strong antioxidant and free radical scavenger activity. These included lactic acid and cholic acid which have been reported to inhibit lipid peroxidation [29,30] as well as vitamin D3 which is known to have anti-inflammatory, anti-oxidative, and anti-peroxidative properties [31]. GT3 is a known antioxidant and free radical scavenger [17,32].

We also observed an increase in the serum levels of docosahexaenoic acid which is an ω-3 fatty acid known for anti-inflammatory activity as well as reduction of thrombosis and platelet activation. These results show that metabolic shifts caused by GT3 augment protection from radiation stress that is known to induce thrombosis, inflammation, and oxidative stress. Remarkably, these changes are accentuated between 1–3 days, suggesting maximal radioprotective efficacy of GT3 is likely during this time frame. Studies in murine and NHP models have demonstrated that GT3 is most effective as a radioprotector when administered 24 h prior to exposure with lethal dose of ionizing radiation [17]. Our metabolomics results corroborate with CBC and blood chemistry results wherein some changes in CBCs and blood chemistry are apparent after 24 h post-GT3 injection and normalize to near normal levels overtime.

We examined the effect of gender on the metabolomic and lipidomic profiles of NHPs following GT3 administration and observed an underlying difference between males and females at baseline. This could be attributed to fundamental physiological differences between the two genders. Importantly, the trend in metabolic alterations in response to GT3 dosing was similar in both genders across the time course that was monitored. The time points with the widest divergence against control showed no significant gender effect on metabolite abundance profiles. Furthermore, among the top 20 validated biomarkers we describe in this paper, only one showed a significant adjusted *p*-value at one time point, 3-deoxyvitamin D3 at day 4. Notably, this significance subsided by the end of the study period. It is also important to note that in these validated biomarkers, there is no significant separation between males and females in the control groups. These findings could be attributed in part to the fact that GT3 administration inherently showed few changes in the dosed animals and hence gender effects may be too subtle to dissect out. However, future investigations from our laboratory will examine the metabolic changes with GT3 dosing in irradiated animals where these differences may become more apparent.

In conclusion, molecular phenotyping performed using global metabolomic and lipidomic studies demonstrate that administration of GT3 does not cause any adverse effects at the molecular level. The serum metabolite profiles provide insights into biochemical alterations caused by GT3 that could contribute to its radioprotection observed in animal models. We plan to carry out comprehensive metabolomic and lipidomic studies with serum samples of NHPs injected with GT3 and exposed to different doses of ionizing radiation.

## 4. Materials and Methods

### 4.1. Animals and Animal Care

Twelve naïve rhesus macaques (*Macaca mulatta*, Chinese sub strain) (six males and six females) 3–5 years of age, weighing 3.5–4.5 kg, were obtained from Primate Products, Inc. (Miami, FL, USA) and maintained in a facility accredited by the Association for Assessment and Accreditation of Laboratory Animal Care (AAALAC)-International. Animals were quarantined for six weeks prior to initiation of the experiment. Animal housing, health monitoring, care, and enrichment during the experimental period have been described earlier [20]. Animals were fed primate diet (Teklad T.2050 diet; Harlan^®^ Laboratories Inc., Madison, WI, USA) twice daily with at least six hours between feedings (animals were fed four biscuits each at 07:00 a.m. and 02:00 p.m.) and received drinking water *ad libitum*. Animals were stratified by gender and body weight during the quarantine period and then assigned to different dose-groups of GT3. Due to study-specific reasons, paired housing was not possible during the experiment. The animals were housed individually, but they were able to see and touch conspecifics through the cage divider. This also eliminated the chance of conflict injuries that could have been caused by pair-housing. All procedures involving animals were approved by the Armed Forces Radiobiology Research Institute Institutional Animal Care and Use Committee (IACUC) (P2010-12-017, 15 April 2011) and Department of Defense Animal Care and Use Review Office (ACURO) (2 May 2011). This study was carried out in strict accordance with the recommendations in the *Guide for the Care and Use of Laboratory Animals of the National Institutes of Health* [33].

### 4.2. Drug Preparation and Administration

Pyrogen-free samples of GT3 formulation (50 mg/mL) in 5% Tween-80 in saline, were purchased from Yasoo Health Inc. (Johnson City, TN, USA). The doses of GT3 for NHP were 9.375, 18.75, and 37.5 mg/kg adjusted precisely to the body weight of individual NHPs. GT3 was administered sc to 12 animals (four animals with each dose) at the dorsal scapular area (between the shoulder blades).

### 4.3. Serum Sample Collection

Blood was collected by venipuncture from the saphenous vein of the lower leg after the site was cleaned using a 70% isopropyl alcohol wipe and dried with sterile gauze. All animals were restrained using the pole-and-collar method and placed in a chair for blood collection. On the day of drug administration, animals were bled repeatedly at 0.25, 0.5, 1, 2, 4, 8, and 12 h post-injection. On days when animals were only bled once, the blood draw was conducted between 08:00 a.m. and 10:00 a.m., 1–3 h after animals were fed. The relation from the time of feeding to any specific bleeding was consistent for all animals. The desired volume of blood was collected with a 3 mL disposable luer-lock syringe with 25-gauge needle. For serum collection, the blood sample was transferred to Capiject serum separator tubes (3T-MG; Terumo Medical Corp, Elkton, MD, USA), and allowed to clot for 30 min, then centrifuged (10 min, 400× *g*). Serum samples were stored at −70 °C until blood biochemistry analysis or shipped on dry ice to the Georgetown University Medical Center for metabolomic study. Blood samples for CBC were collected in EDTA (ethylenediaminetetraacetic acid) blood collection tubes (Sarstedt Inc., Newton, NC, USA) and mixed in a rotary shaker. 

### 4.4. Serum Metabolomics Using UPLC QTOF Analysis

Serum was prepared for metabolomic analysis as described previously [34,35]. Briefly, metabolite extraction was performed by adding 75 μL of 40% isopropanol (IPA) + 25% methanol + 35% water containing internal standards to 25 μL of NHP plasma. Samples were vortexed and incubated on ice for 20 min. One hundred μL of 100% acetonitrile (ACN) was then added to samples. Vials were incubated at −20 °C for 15 min and centrifuged at 13,000 rpm at 4 °C for 20 min. Supernatant was transferred to fresh vials for UPLC-ESI-Q-TOF-MS analysis.

For metabolomic analysis, each sample (2 μL) was injected onto a reverse-phase 50 × 2.1 mm Acquity 1.7-μm BEH C18 column at 60 °C column temperature (Waters Corp, Milford, MA, USA) using an Acquity UPLC system (Waters) with a gradient mobile phase consisting of 100% water containing 0.1% formic acid (Solvent A) and 100% ACN containing 0.1% formic acid (Solvent B) and 90% IPA + 10% ACN containing 0.1% formic acid (Solvent C), and resolved for 13 min at a flow rate of 0.5 mL/min. The gradient started with 98% A and 2% B for 0.5 min with a ramp of curve 6. At 4 min, the gradient reached 40% A and 60% B. At 8 min, the gradient shifted to 2% A and 98% B for one min. From 9.5 to 11 min, the gradient was 98% C and 2% B. At 11.50 min, it shifted to 50% A and 50% B. At 12 min, it reached initial conditions. In addition, for lipidomic analysis, samples were run on a reverse-phase 100 × 2.1 mm Acquity 1.7-μm CSH C18 column (Waters Corp, Milford, MA, USA). The run time was 11 min at a flow rate of 0.45 mL/min at a column temperature of 65 °C. The gradient included 2 solvents: 50% ACN + 50% water with 0.1% formic acid and 10 mM ammonium formate (Solvent A) and 90% IPA + 10% ACN containing 0.1% formic acid and 10 mM ammonium formate (Solvent B). The gradient started with 60% A and 40% B with a ramp of curve 6 for 0.5 min. At 8 min, the gradient reached 100% B. At 9 min, the gradient returned to initial conditions.

The column eluent was introduced directly into the mass spectrometer by electro-spray. Mass spectrometry was performed on a Q-TOF MS (Xevo G2 QTOF MS, Waters Corporation, Milford, MA, USA), operating in either negative-ion (ESI−) or positive-ion (ESI+) electro-spray ionization mode with a capillary voltage of 3 kV for positive mode and 1.5 kV for negative mode and a sampling cone voltage of 30 V in both negative and positive modes. The extraction cone was 3.0. The desolvation gas flow was set to 1000 L/h and the temperature was set to 500 °C. The cone gas flow was 25 L/h, and the source temperature was 120 °C. Accurate mass was maintained by introduction of LockSpray interface of Leucine Enkephalin (556.2771 [M + H]+ or 554.2615 [M − H]−) at a concentration of 2 ng/μL in 50% aqueous ACN and a rate of 5 μL/min. Data were acquired in centroid mode from 50 to 1200 *m*/*z* in MS scanning. Pooled QC (quality control samples) were run throughout the batch to monitor data reproducibility.

### 4.5. Analysis for CBC and Blood Biochemistry

Total white blood cells (WBC), erythrocytes (red blood cells (RBC)), platelets, neutrophils, lymphocytes, monocytes, reticulocytes, basophils, hemoglobin (HGB), and hematocrit (HCT) were counted using an Advia 120-cell counter (Bayer Corporation, Tarrytown, NY, USA) [20]. Blood biochemistry parameters were investigated using Vitros 350 Chemistry System (Ortho Clinical Diagnostics, Raritan, NJ, USA) [20]. Though 23 parameters were analyzed, we have presented the results of eight parameters (glucose, albumin, ALT (alanine aminotransferase), AST (aspartate aminotransferase), ALKP (alkaline phosphatase), total bilirubin, total protein, and GGT (gamma-glutamyl transferase)) where we notice changes in response to GT3 administration.

### 4.6. Data Processing and Statistical Analysis

Centroided and integrated mass spectrometry data from the UPLC-TOFMS were preprocessed using XCMS software (Scripps Research Institute, La Jolla, CA, USA) to generate a data matrix containing ion intensities, mass to charge (*m*/*z*) and retention time values. The data were normalized to the intensities of internal standards. Multivariate statistics were performed (log transformed and Pareto scaled) using Metaboanalyst V3.0 (Xia Lab, McGill University, Montreal, QC, Canada) and R scripts developed in-house. An ANOVA comparison was used to identify significantly dysregulated metabolites (based on *m*/*z* values) between comparative groups. The identity of these metabolites was confirmed using tandem mass spectrometry. Additionally, the identity of lipids were confirmed by using the SIMPLIPID software V6.01 (Premier Biosoft, Palo Alto, CA, USA), by fragmentation pattern matching. Two-tailed t-tests and ANOVA comparisons were performed in R to show significance in gender and dose comparisons, respectively. Pathway analysis was performed using the Ingenuity pathway analysis (IPA) software (Qiagen, Germantown, MD, USA). We applied a multiple testing correction using Bonferroni method.

For CBC and blood biochemistry data, mean values with standard errors (SE, when applicable) are reported. Paired sample *t*-tests were used to detect if there were significant differences between pre- and post-GT3 injection time points. All statistical tests were two-sided, with a 5% significance level. Statistical software SPSS version 22 (IBM, Armonk, NY, USA) and GraphPad Prism 5 (GraphPad Software, Inc., La Jolla, CA, USA) were used for analyses.

## Supplementary Materials

Supplementary materials can be found at www.mdpi.com/1422-0067/19/1/79/s1.

## References

[1] Health and Human Services HHS Enhances Nation’s Health Preparedness for Radiological Threats. https://www.hhs.gov/about/news/2016/10/06/hhs-enhances-nation-s-health-preparedness-radiological-threats.html.

[2] Gosden C., Gardener D. (2005). Weapons of mass destruction--threats and responses. BMJ.

[3] Singh V.K., Seed T.M. (2017). A review of radiation countermeasures focusing on injury-specific medicinals and regulatory approval status: Part I. Radiation sub-syndromes, animal models and FDA-approved countermeasures. Int. J. Radiat. Biol..

[4] Seed T.M. (2005). Radiation protectants: Current status and future prospects. Health Phys..

[5] Hall E.J., Giaccia A.J. (2012). Radiobiology for the Radiologist.

[6] DiCarlo A.L., Maher C., Hick J.L., Hanfling D., Dainiak N., Chao N., Bader J.L., Coleman C.N., Weinstock D.M. (2011). Radiation injury after a nuclear detonation: Medical consequences and the need for scarce resources allocation. Disaster Med. Public Health Prep..

[7] Waselenko J.K., MacVittie T.J., Blakely W.F., Pesik N., Wiley A.L., Dickerson W.E., Tsu H., Confer D.L., Coleman C.N., Seed T. (2004). Medical management of the acute radiation syndrome: Recommendations of the Strategic National Stockpile Radiation Working Group. Ann. Intern. Med..

[8] Farese A.M., MacVittie T.J. (2015). Filgrastim for the treatment of hematopoietic acute radiation syndrome. Drugs Today.

[9] National Institute of Allergic and Infectious Diseases Pegfilgrastim Approved for Treating Acute Radiation Syndrome. https://www.niaid.nih.gov/topics/radnuc/Pages/pegfilgrastim.aspx.

[10] U.S. Food and Drug Administration FDA Approves Neupogen for Treatment of Patients with Radiation-Induced Myelosuppression Following a Radiological/Nuclear Incident. http://www.fda.gov/EmergencyPreparedness/Counterterrorism/MedicalCountermeasures/AboutMCMi/ucm443245.htm.

[11] Pellmar T.C., Rockwell S. (2005). Priority list of research areas for radiological nuclear threat countermeasures. Radiat. Res..

[12] Krivokrysenko V.I., Toshkov I.A., Gleiberman A.S., Krasnov P., Shyshynova I., Bespalov I., Maitra R.K., Narizhneva N.V., Singh V.K., Whitnall M.H. (2015). The Toll-like receptor 5 agonist Entolimod mitigates lethal acute radiation syndrome in non-human primates. PLoS ONE.

[13] Gluzman-Poltorak Z., Mendonca S.R., Vainstein V., Kha H., Basile L.A. (2014). Randomized comparison of single dose of recombinant human IL-12 versus placebo for restoration of hematopoiesis and improved survival in rhesus monkeys exposed to lethal radiation. J. Hematol. Oncol..

[14] Humanetics Pharmaceuticals BIO 300: Development Programs. http://humanetics.a03.neon.ittrium.com/development-programs.

[15] Sen C.K., Khanna S., Roy S. (2006). Tocotrienols: Vitamin E beyond tocopherols. Life Sci..

[16] Singh V.K., Beattie L.A., Seed T.M. (2013). Vitamin E: Tocopherols and tocotrienols as potential radiation countermeasures. J. Radiat. Res..

[17] Singh V.K., Hauer-Jensen M. (2016). γ-tocotrienol as a promising countermeasure for acute radiation syndrome: Current status. Int. J. Mol. Sci..

[18] Meganathan P., Jabir R.S., Fuang H.G., Bhoo-Pathy N., Choudhury R.B., Taib N.A., Nesaretnam K., Chik Z. (2015). A new formulation of Gamma Delta Tocotrienol has superior bioavailability compared to existing Tocotrienol-Rich Fraction in healthy human subjects. Sci. Rep..

[19] Kulkarni S., Singh P.K., Ghosh S.P., Posarac A., Singh V.K. (2013). Granulocyte colony-stimulating factor antibody abrogates radioprotective efficacy of gamma-tocotrienol, a promising radiation countermeasure. Cytokine.

[20] Singh V.K., Kulkarni S., Fatanmi O.O., Wise S.Y., Newman V.L., Romaine P.L., Hendrickson H., Gulani J., Ghosh S.P., Kumar K.S. (2016). Radioprotective efficacy of gamma-tocotrienol in nonhuman primates. Radiat. Res..

[21] U.S. Food and Drug Administration Guidance for Industry: Product Developoment under the Animal Rule. http://www.fda.gov/downloads/Drugs/GuidanceComplianceRegulatoryInformation/Guidances/UCM399217.pdf.

[22] Berbee M., Fu Q., Boerma M., Sree Kumar K., Loose D.S., Hauer-Jensen M. (2012). Mechanisms underlying the radioprotective properties of gamma-tocotrienol: Comparative gene expression profiling in tocol-treated endothelial cells. Genes Nutr..

[23] Berbee M., Fu Q., Boerma M., Pathak R., Zhou D., Kumar K.S., Hauer-Jensen M. (2011). Reduction of radiation-induced vascular nitrosative stress by the vitamin E analog gamma-tocotrienol: Evidence of a role for tetrahydrobiopterin. Int. J. Radiat. Oncol. Biol. Phys..

[24] Berbee M., Fu Q., Boerma M., Wang J., Kumar K.S., Hauer-Jensen M. (2009). Gamma-Tocotrienol ameliorates intestinal radiation injury and reduces vascular oxidative stress after total-body irradiation by an HMG-CoA reductase-dependent mechanism. Radiat. Res..

[25] Fendler W., Malachowska B., Meghani K., Konstantinopoulos P.A., Guha C., Singh V.K., Chowdhury D. (2017). Evolutionarily conserved serum microRNAs predict radiation-induced fatality in nonhuman primates. Sci. Transl. Med..

[26] Pannkuk E.L., Laiakis E.C., Fornace A.J., Fatamni O.O., Singh V.K. (2017). A metabolomic serum signature from nonhuman primates treated with a radiation countermeasure, gamma-tocotrienol, and exposed to ionizing radiation. Health Phys..

[27] Pannkuk E.L., Fornace A.J., Laiakis E.C. (2017). Metabolomic applications in radiation biodosimetry: Exploring radiation effects through small molecules. Int. J. Radiat. Biol..

[28] Chen Z., Coy S.L., Pannkuk E.L., Laiakis E.C., Hall A.B., Fornace A.J., Vouros P. (2016). Rapid and high-throughput detection and quantitation of radiation biomarkers in human and nonhuman primates by differential mobility spectrometry-mass spectrometry. J. Am. Soc. Mass Spectrom..

[29] Groussard C., Morel I., Chevanne M., Monnier M., Cillard J., Delamarche A. (2000). Free radical scavenging and antioxidant effects of lactate ion: An in vitro study. J. Appl. Physiol..

[30] DeLange R.J., Glazer A.N. (1990). Bile acids: Antioxidants or enhancers of peroxidation depending on lipid concentration. Arch. Biochem. Biophys..

[31] Ke C.-Y., Yang F.-L., Wu W.-T., Chung C.-H., Lee R.-P., Yang W.-T., Subeq Y.-M., Liao K.-W. (2016). Vitamin D(3) reduces tissue damage and oxidative stress caused by exhaustive exercise. Int. J. Med. Sci..

[32] Palozza P., Verdecchia S., Avanzi L., Vertuani S., Serini S., Iannone A., Manfredini S. (2006). Comparative antioxidant activity of tocotrienols and the novel chromanyl-polyisoprenyl molecule FeAox-6 in isolated membranes and intact cells. Mol. Cell. Biochem..

[33] National Research Council of the National Academy of Sciences (2011). Guide for the Care and Use of Laboratory Animals.

[34] Kaur P., Rizk N., Ibrahim S., Luo Y., Younes N., Perry B., Dennis K., Zirie M., Luta G., Cheema A.K. (2013). Quantitative metabolomic and lipidomic profiling reveals aberrant amino acid metabolism in type 2 diabetes. Mol. Biosyst..

[35] LaConti J.J., Laiakis E.C., Mays A.D., Peran I., Kim S.E., Shay J.W., Riegel A.T., Fornace A.J., Wellstein A. (2015). Distinct serum metabolomics profiles associated with malignant progression in the Kras(G12D) mouse model of pancreatic ductal adenocarcinoma. BMC Genom..

