# Supplementary material for: A Metabolomic and Lipidomic Serum Signature from Nonhuman Primates Administered with a Promising Radiation Countermeasure, Gamma-Tocotrienol
Source: Int J Mol Sci. 2017 Dec 28;19(1):79. doi: 10.3390/ijms19010079 (PMC5796029; doi:10.3390/ijms19010079)
Supplement: Supplementary file 1 [file ijms-19-00079-s001.zip › Supplemental Figures S1-5.pdf]

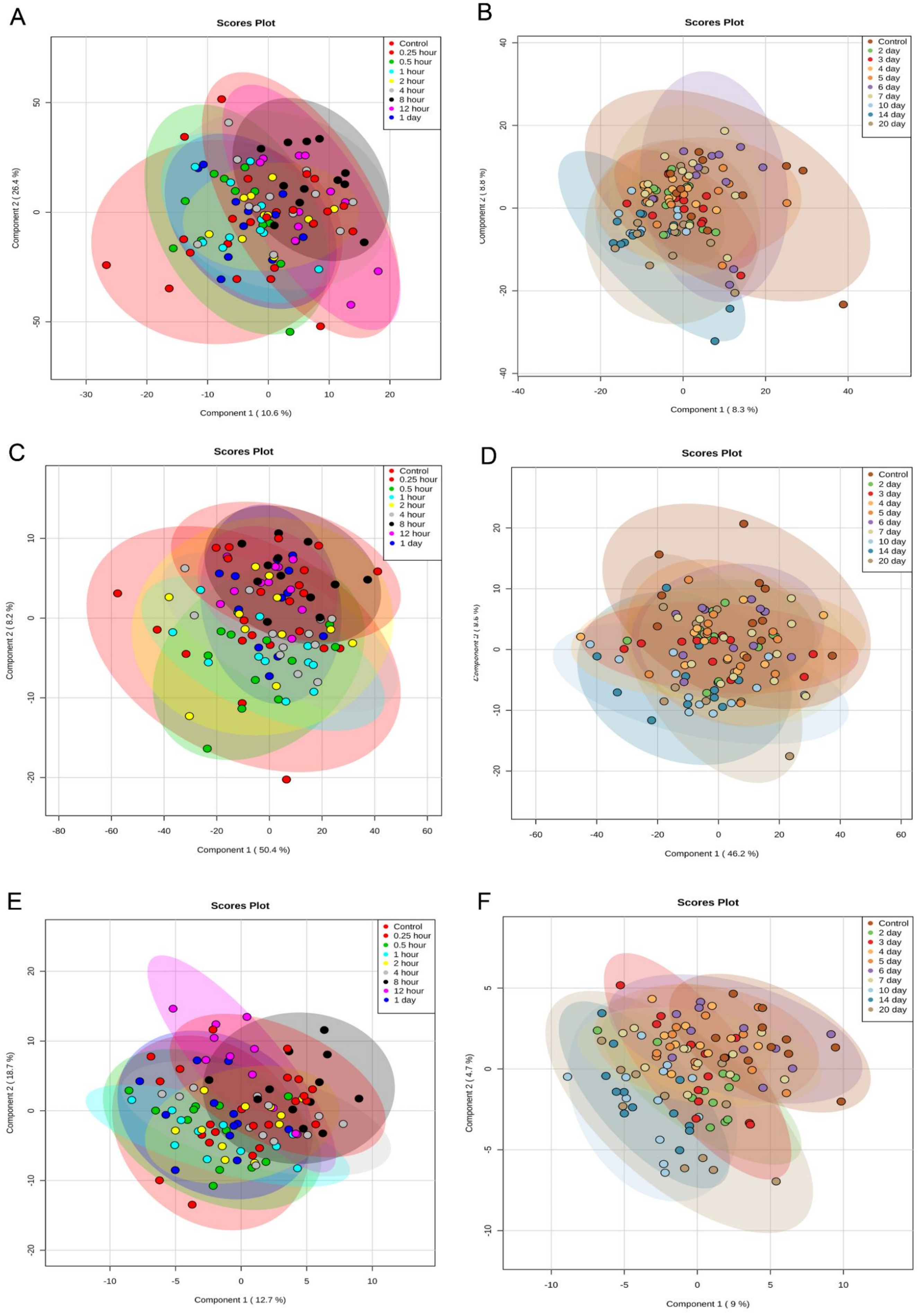

Supplementary Figure S1. Multivariate analysis showing metabolic profiles across all GT3-treated NHPs: (**A**) short term (first 24 h) metabolomics negative, (**B**) long-term (2 days to 20 days) metabolomics negative, (**C**) short term lipidomics positive (**D**) long term lipidomics positive (**E**) short term lipidomics negative and (**F**) long term lipidomics negative. All groups demonstrated minimal separation, indicating no significant changes.

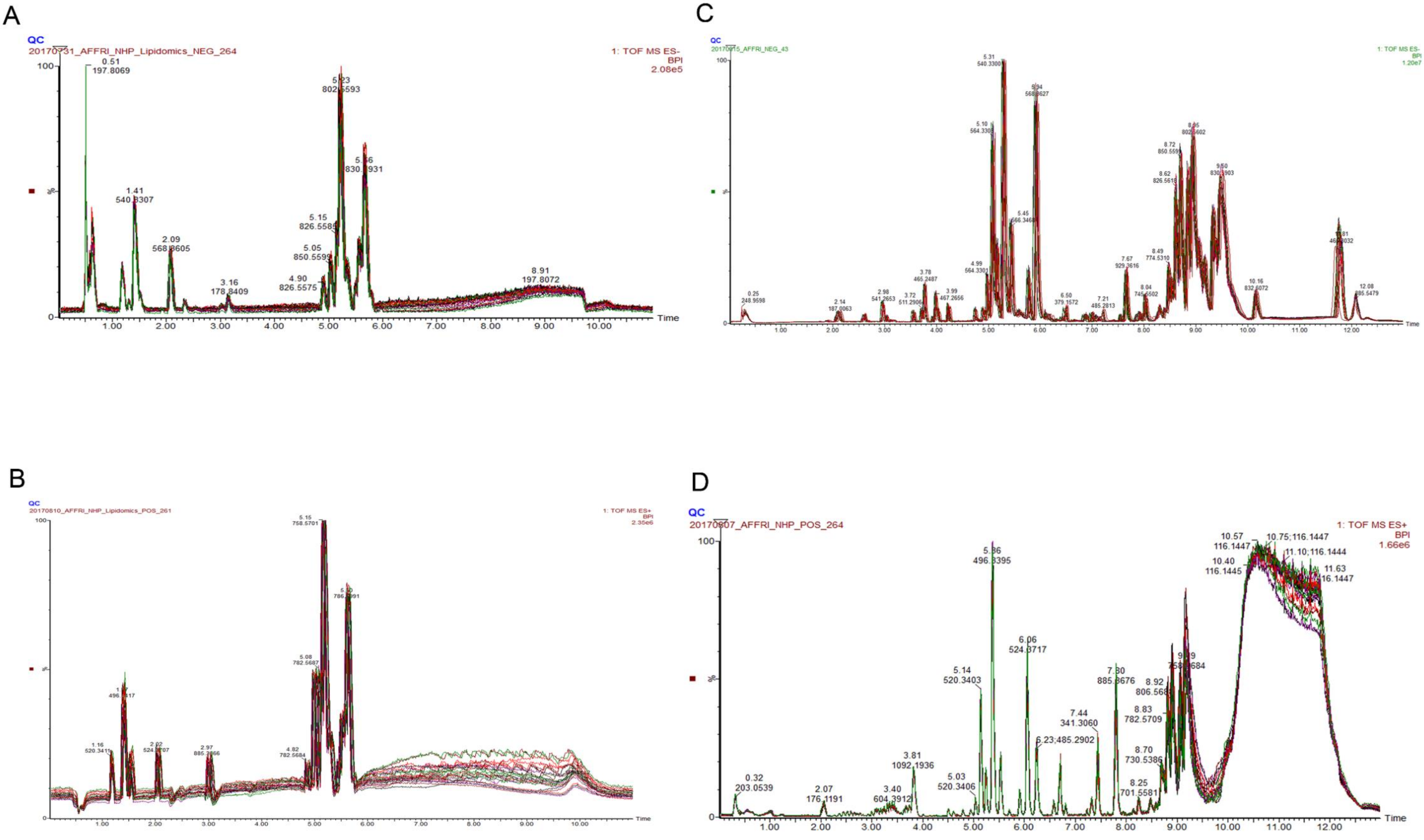

Supplementary Figure S2. TIC overlays for QC samples in (**A**) lipidomics negative, (**B**) lipidomics positive, (**C**) metabolomics negative, and (**D**) metabolomics positive with internal standard coefficient of variance of 5.8%, 18.05%, 5.66% and 25.48%, respectively.

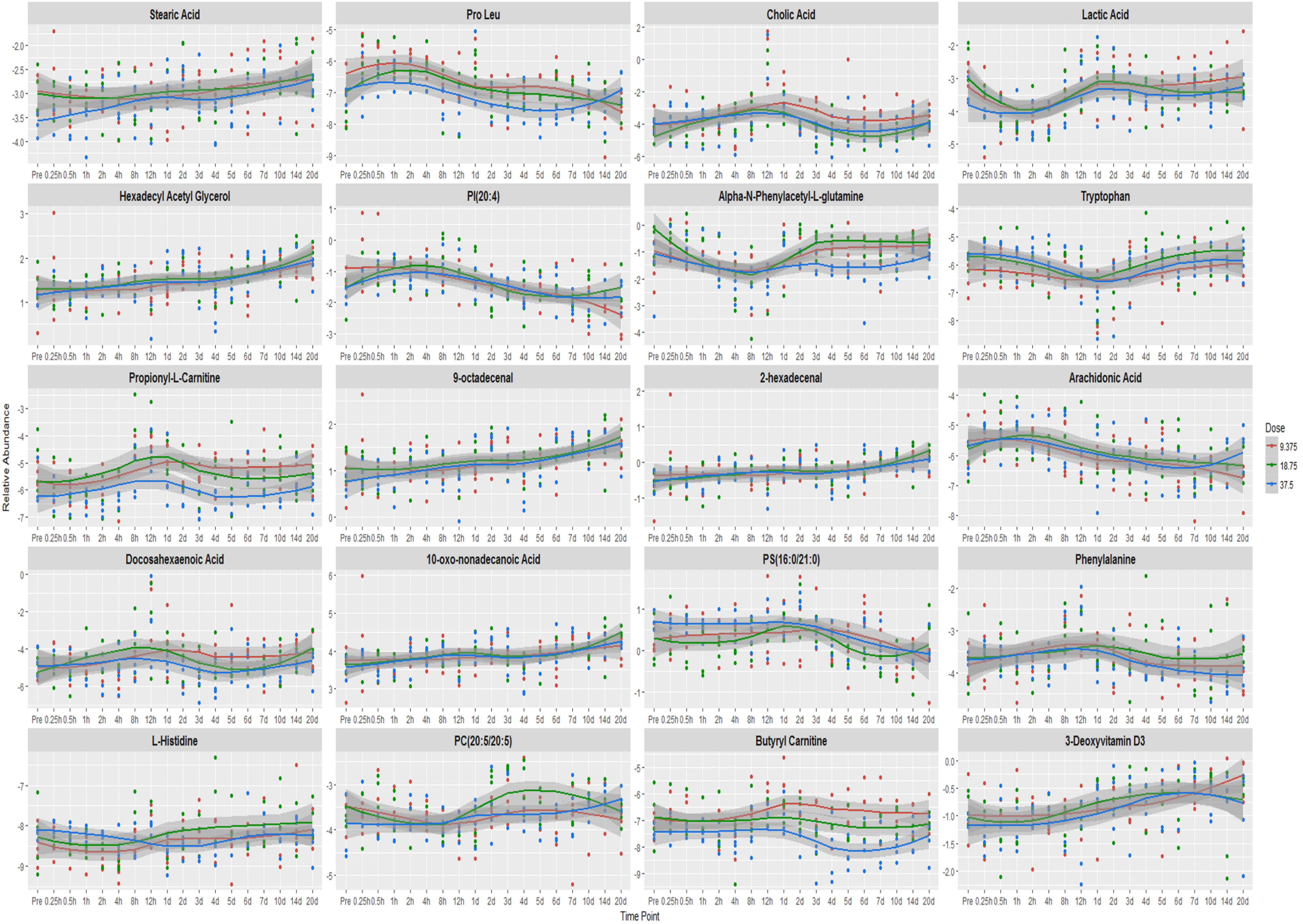

Supplementary Figure S3. Trend lines for top 20 dysregulated biomarkers, for each GT3 dose.

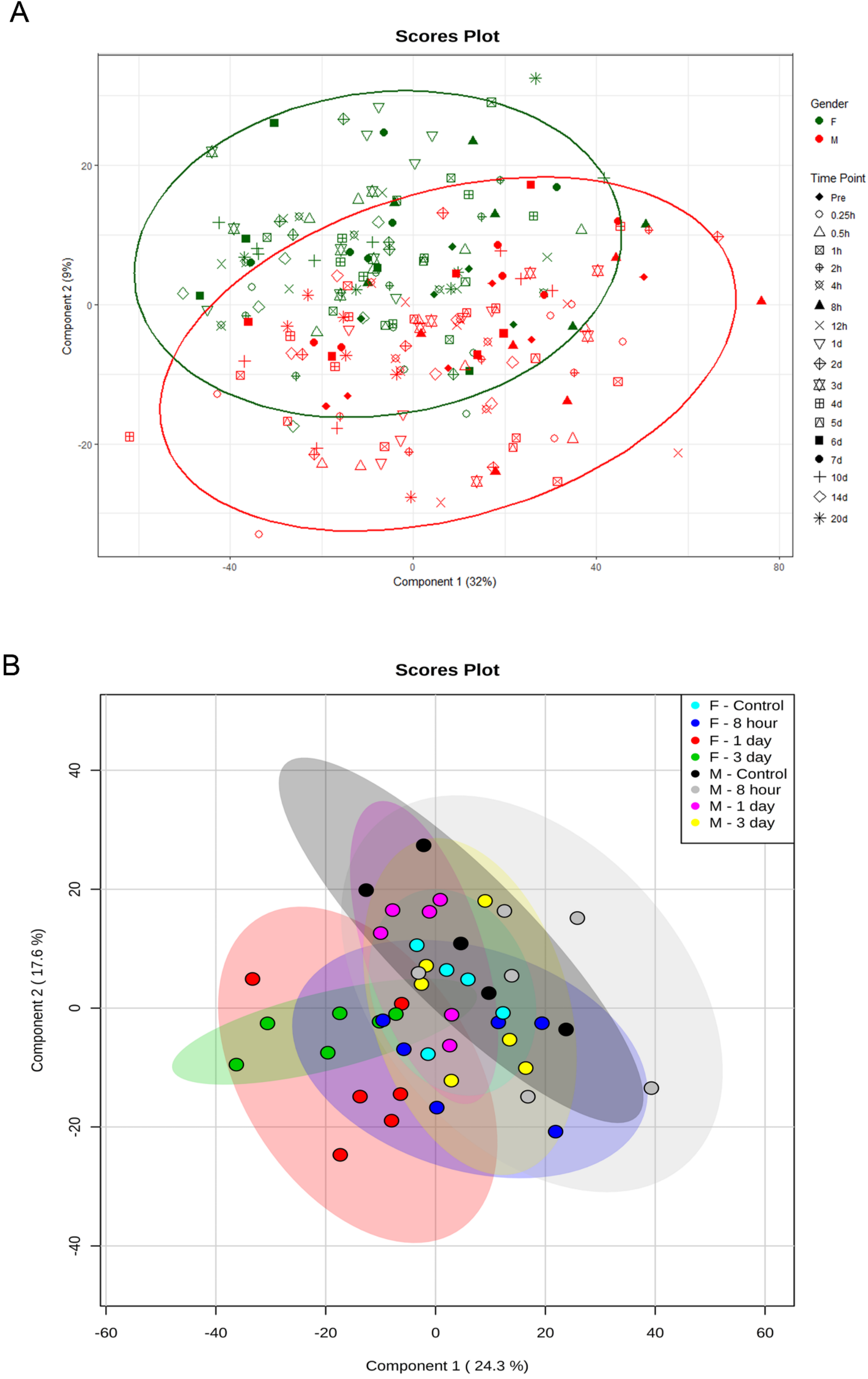

Supplementary Figure S4. Multivariate analysis showing metabolic profiles (**A**) across all GT3-treated NHPs, separated by gender and (**B**) A subset of time points that were shown to have maximal separation against control.

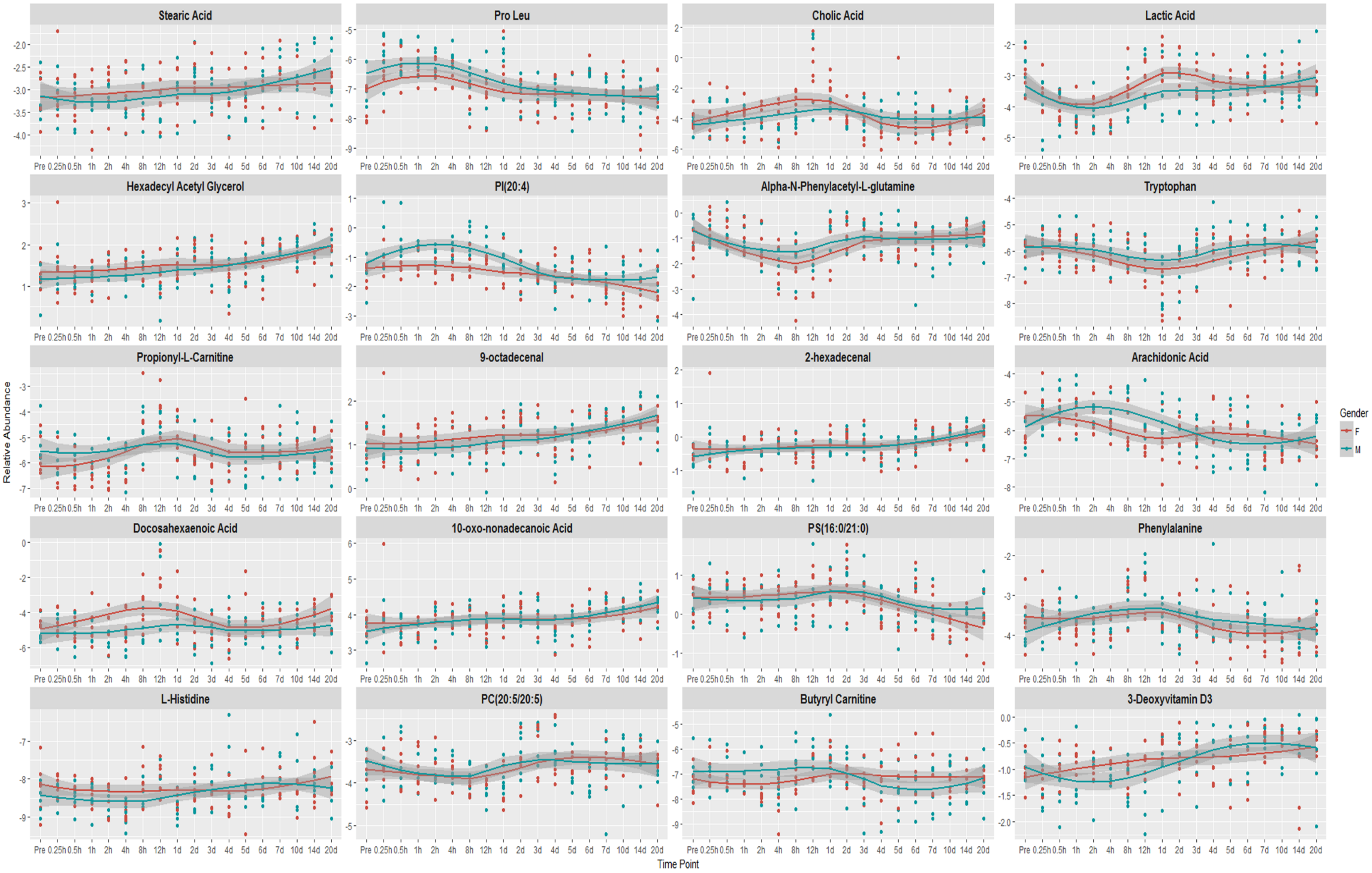

Supplementary Figure S5. Trend lines for top 20 dysregulated biomarkers delineated by gender.
